# The Effect of Cationic Charge Density Change on Transfection Efficiency of Polyethylenimine

**Published:** 2013-02

**Authors:** Zohreh Rezvani Amin, Mohammad Rahimizadeh, Hossein Eshghi, Ali Dehshahri, Mohammad Ramezani

**Affiliations:** 1 Department of Chemistry, School of Sciences, Ferdowsi University of Mashhad, Mashhad, Iran; 2 Department of Pharmaceutical Biotechnology and Pharmaceutical Sciences Research Centre, Faculty of Pharmacy, Shiraz University of Medical Sciences, Shiraz, Iran; 3 Pharmaceutical Research Centre, School of Pharmacy, Mashhad University of Medical Sciences, Mashhad, Iran

**Keywords:** Gene Transfer Technique, Genetic vector, Nanoparticles, Polyethylenimine, Toxicity

## Abstract

***Objective(s):*** Polyethylenimine (PEI) is a potent non-viral gene delivery carrier. PEI surface charge plays a major role in its condensation ability which in turn is a necessary requirement for high transfection efficiency. As the PEI charge density is dependent on pH, the effect of pH changing PEI on PEI condensation ability, transfection efficiency, and cytotoxicity was investigated.

***Materials and Methods:*** In order to compare the different values of pH, 25 kDa PEI solutions were prepared at the pH values of 5, 7 and 10, afterward complexed with two plasmids encoding reporter genes of luciferase and enhanced green fluorescence protein to evaluate the capability of polymers in plasmid delivery at two incubation time (4 versus 24 hr).

***Results:*** The condensation ability of PEI in acidic, neutral and basic environments was similar. At low pH value, the polyplex had negative surface charge whereas at a higher pH value, the surface charge increased and became positive. The higher transfection efficiency and lower cytotoxicity were achieved by the polyplexes prepared at the pH value of 5 with longer incubation time of 24 hr.

***Conclusion: ***The results showed the impact of pH value of PEI on biological and biophysical properties of polyplexes. Although the proton sponge effect can justify several properties of PEI, these results suggest that the proton sponge hypothesis may not be applicable for polymers with low buffering capacity at the pH values around 5.

## Introduction

In recent decades, gene therapy has become a potentially promising strategy to treat acquired or hereditary diseases. Although the majority of gene delivery systems are based on viral carriers, the immunologic responses, potential oncogenicity, limited loading capacity of genetic material and difficult production has limited their wide applications (1). These concerns have stimulated researchers to apply non-viral gene delivery systems which show several advantages due to less cytotoxicity, easy production and modification as well as stability (2, 3). PEI is a potent non-viral gene carrier which exhibits high transfection efficiency (2). Wide range application of PEI is still limited because of the relatively low transfection efficiency when compared to viral delivery systems as well as cytotoxic effects (4, 5). High positive charge on the surface of PEI leads to electrostatic interaction with the negatively charged components of the cell membrane and causes membrane disruption and damage (6). Different approaches have been employed to reduce PEI cytotoxicity by decreasing the positive charge density of the polymer such as acetylation (7, 8), carboxyalkylation of primary amines (9), conjugation of succinate, butanoate and hexanoate to PEI (10, 11). Likewise, several approaches such as pegylation have been studied in order to change the non-specific electrostatic polyplex interaction, inhibiting activation of the reticuloendothelial system, and increasing the half-life of polyplexes in blood (12- 14). It has been proven that the susceptibility of these shielded polyplexes to aggregation induced by salt has been reduced considerably (12). On the other hand, one of the most important properties of PEI is its high cationic charge density. Every third atom of PEI is protonable nitrogen which confers significant buffering capacity over a wide pH range. The theoretical ratio of primary, secondary and tertiary amino groups in PEI has been calculated as 1:2:1, respectively. The high charge density of this polymer is as a result of the protonation of amino groups in the biological environments. Therefore, there is a relationship between the pH of PEI and the positive charge density on PEI (3). In former investigations, it has been revealed that the PEI protonation degree at pH value of 7.4 is 20% which increased to 45% at pH value of 5 (15, 16). Recently, a cost-effective gene transfection by DNA compaction at pH 4 using PEI has been reported (17). In the present study, we assumed that increasing the positive charge density on PEI by reducing the pH of PEI may have an impact on the biophysical and biological properties of PEI. To address the impact of pH change on condensation ability, particle size, zeta potential, transfection time and cytotoxicity, as well as transfection efficiency of PEI/plasmid DNA complexes prepared at different pH values were studied.

## Materials and Methods

*Materials*

Branched PEI (average MW 25 kDa) was purchased from Polyscience, Inc (Warrington, PA). Plasmid pRL-CMV-luc (Renilla luciferase under control of the cytomegalovirus (CMV) enhancer/promoter), luciferase assay kit and CellTiter 96® AQueous non-radioactive cell proliferation assay (MTT) were purchased from Promega (Madison, WI, USA). Ethidium bromide was obtained from Cinnagen (Tehran, Iran). All solvents and chemicals were obtained from Sigma-Aldrich (Munich, Germany) and were of the highest purity available. Dialyses were carried out using Spectra/Por dialysis membranes (Spectrum Laboratories, Houston, TX USA).

*Preparation of PEI solutions at various pHs*

PEI solutions were prepared in double distilled water (DDW). The pH value of each solution was adjusted to 5, 7, and 10 using HCl (1N) and measured with a pH meter (Mettler Todelo, Greifensee, Switzerland). Resultant NaCl was removed using a dialysis membrane (10000 cut off, spectra/por membrane) against DDW for 18 hr, then pH measured again and adjusted at the desired values. Finally the solutions were lyophilized to obtain a powder for further experiments. 

*Preparation of plasmid DNA*

pRL-CMV-luc was transformed into *Escherichia coli *strain DH5α, propagated in selective Luria–Bertani (LB) medium, centrifuged and extracted from the cell pellets using the Qiagen Endofree Mega Plasmid Kit (QIAGEN, Hilden, Germany) according to the manufacturer’s instructions. The purity of the plasmid DNA was measured on a UV spectrophotometer using A260/A280 ratios.

*Measurement of PEI binding strength by ethidium bromide (EtBr) exclusion assay*

EtBr intercalation in pDNA was determined by fluorescence spectroscopy (excitation: 510 nm and emission: 590 nm) measured in a Jasco FP-6200 spectrofluorometer (Tokyo, Japan). A solution of PEI in DDW was added stepwise to a solution of plasmid DNA (5 µg/ml) and EtBr (0.4 mg/ml) in HBG buffer (20 mM Hepes, 5.2% glucose, pH 7.0), and fluorescence intensity was measured. The fluorescence intensity of the EtBr solution in the presence of free plasmid corresponds to 0% condensation, and the fluorescence intensity without plasmid corresponds to 100% condensation. All measurements were performed in triplicate by spectrofluorometer and the graph was constructed by plotting the relative fluorescence intensity (%) against the polymer/plasmid DNA ratio (w/w). Preparation of the DNA condensing activity was estimated graphically by plotting relative fluorescence intensity (%) against the PEI /plasmid DNA ratio (w/w). 

*Gel retardation assay*

Solutions of PEI at different pH values prepared in DDW and added to a solution of DNA (3 μg) in DDW for preparation of the desired PEI/DNA weight ratios. The mixture were incubated at room temperature for 30 min, afterward loading dye was added, and 10 μl of the solution was run on a 1% agarose gel (100 V, 90 min). DNA was visualized with ethidium bromide and the bands corresponding to plasmid DNA and DNA/nanoparticle complexes were visualized by an ultraviolet (UV) illuminator. 

*Particle size and zeta potential measurements*

The mean hydrodynamic particle size and charge measurements for PEI/pDNA complexes were performed using Dynamic Light Scattering (DLS) and Laser Doppler Velocimetry (LDV), respectively, using Malvern Nano ZS instrument and DTS software (Malvern Instruments, UK) in DDW. Various amounts of cationic polymers were diluted in 125 µl of DDW and mixed with an equal volume of DDW containing DNA. The measurements were carried out in automatic mode and the results are presented as mean ± SD. Each mean represents the average value of 3 measurements.

*Cell line and cell culture*

Neuro2A murine neuroblastoma cells (ATCC CCL-131) were grown in DMEM (1 g/l glucose, 2 mM glutamine) supplemented with 10% FBS, streptomycin at 100 µg/ml and penicillin at 100 U/ml. All cells were incubated at 37°C in a humidified 5% CO_2_ atmosphere.

*Transfection procedure*

Cells were seeded at a density of 1×10^4^ cells/well in 96-well plates 1 day prior to transfection experiments, and grown in the appropriate medium with 10% fetal bovine serum. Different polymer/plasmid DNA weight ratios (C/P or w/w) were used to prepare the polycation/plasmid complexes (i.e. polyplexes). This mixture was added to the wells of 96-well plates containing 60–90% confluent cultures of cells in medium with no FBS and allowed to incubate for 4 hr. Subsequently, the medium was replaced with a fresh complete medium containing 10% FBS and gene expression was assayed 24 hr later. In another experiment, the cells were exposed to polyplexes for 24 hr where polyplexes were added to the cells in medium with no FBS and incubated for 4 hr followed by addition of FBS to achieved concentration of 10% without changing the medium and gene expression was assayed 24 hr later by Promega Renilla Luciferase Assay kit and protocol (Madison, WI) and a luminometer (Berthold). The results were presented as relative light units (RLU) of seeded cells.

*Luciferase reporter gene expression*

Twenty four hr post transfection, the medium was removed and cells were lysed by adding 50 μl of cell lysis buffer (Promega, Madison, WI). Luciferase activity was measured using the Promega *Renilla *Luciferase Assay kit and protocol (Madison, WI) and a luminometer (Berthold Detection Systems, Pforzheim, Germany). The results were revealed as relative light units (RLU) of seeded cells.

*Cell transfection with ρEGFP*

Transfection procedure with pEGFP plasmid was carried out by the same method used for ρRL­CMV plasmid. Reporter gene expression was analyzed using a fluorescent microscopy (JULI Smart Fluorescent Cell Analyzer, NanoEnTek, South Korea).

*Cytotoxicity assay*

The toxicity of complexed PEI with plasmid DNA was evaluated using MTT assay. Neuro2A cell line was cultured in 96-well plates at 1×10^4^ cells per well for 24 hr then treated with the same amounts of polyplexes used for transfection experiments. After 4 hr, the medium was removed and replaced with fresh complete growth media and cultured for additional 24 hr. In another experiment, the cells were exposed to polyplexes for 24 hr where polyplexes were added to the cells in medium with no FBS and incubated for 4 hr followed by addition of FBS to achieve concentration of 10% without changing the medium and cytotoxicity was assayed 24 hr later. MTT reagent (10 μl) was added to each well and incubated for 4 hr at 37°C and then MTT-containing medium was aspirated off and 100 μl of DMSO was added to dissolve the formazan crystal formed by live cells. Absorbance was measured at 570 nm. The cell viability (%) relative to control wells not treated with polyplexes was calculated by [A] test/ [A] control × 100.

## Results

*Biophysical properties of polyplexes*

**Figure 1:**
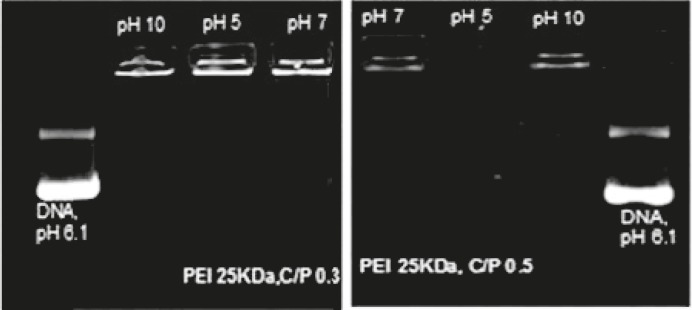
Agarose Gel Electrophoreses of Polyplexes Formed With PEI 25 kDa at C/P Ratio of 0.3 (Left) and 0.5 (Right) at Different pHs

Electrostatic interactions between PEI nitrogen atoms and plasmid phosphate groups result in polyplex formation (18). To investigate the ability of PEI solutions with different pH values to condense plasmid DNA into nanosized particles, the ethidium bromide (EtBr) exclusion assay was carried out. The results of gel retardation assay showed that all polyplexes are able to completely retard the mobility of plasmid DNA at C/P≥1 (data did not presented). To investigate the lower C/P ratios, PEIs were prepared at C/P ratios of 0.3 and 0.5. As revealed in Figure 1, the PEI at pH value of 10 completely retarded plasmid mobility at C/P ratio of 0.3 whereas the PEI at pH value of 7 and 5 showed complete retardation at C/P ratio of 0.5. It is suggested that the reduction of PEI binding affinity at pH values of 5 and 7 (C/P ratio of 0.3) could be a result of acidic pH of plasmid DNA solution (pH= 6.1).

**Figure 2:**
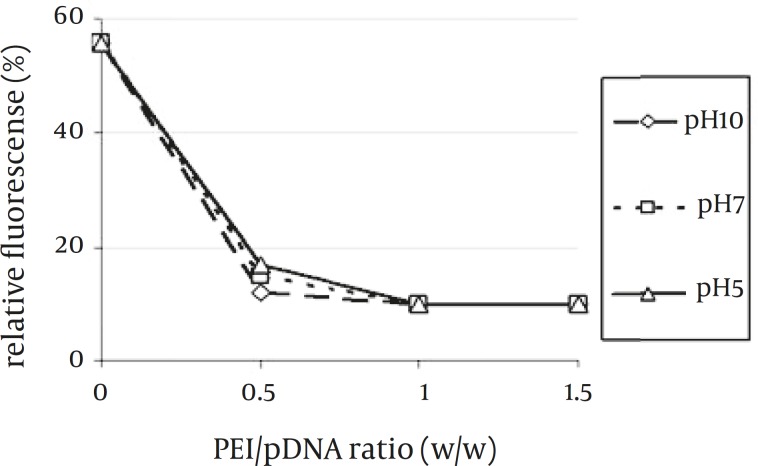
Plasmid DNA Condensation by 25 kDa PEI at Different pH Values Measured by the Ethidium Bromide Exclusion Assay

The ethidium bromide (EtBr) exclusion assay was carried out to investigate the ability of PEI to condense plasmid DNA into nanoparticles (19). DNA intercalation results in a significant increase in fluorescence intensity. Some of the intercalated EtBr is displaced following the PEI binding to the plasmid resulting in measurable reduction in the fluorescence intensity. As shown in Figure 2, all PEI samples at different pH values were able to condense plasmid DNA at C/P ratio of 0.5 completely. 

*Particle size and zeta potential measurements*

Table 1 summarizes the particle size and zeta potential for all the polyplexes at various pH and C/P ratios. At the pH value of 5, the polyplexes surface negative charge was enhanced by increasing the C/P ratio from 0.3 to 2 whereas the surface negative charge decreased following the addition of more polymer at C/P ratios ranging from 2:1 to 6:1. The polyplexes at pH of 10 (C/P ratios of 4 and 6) indicated the highest amounts of surface positive charge. The particles size decreased by increasing the C/P ratio from 0.3 to 2 at pHs of 10 and 5. The polyplexes at pH of 5 (C/P ratios of 4 and 6) revealed the more appropriate size range for transfection experiments.

**Table 1:** Result of Zeta Potential (mV ± SD) and Size of DNA/polymer Complex (nm ± SD)

pH	C/P ratio	Size ± SD (nm)	Zeta Potentioal (mV ± SD)
10	0.3	653±162	7.5±2.2
	0.5	265±28	15.3±0.4
	2	256±19	23.4±4.5
	4	299±15	28.0±3.9
	6	541±67	36.7±2.1
7	0.3	405±30	-2.3±0.1
	0.5	703±40	-3.5±0.2
	2	435±32	-15.2±1.8
	4	906±68	-9.7±1.2
	6	711±53	-9.0±3.4
5	0.3	386±64	-1.58±0.1
	0.5	585±32	-5.9±0.8
	2	753±110	-27±4.3
	4	203±20	-15±3.9
	6	212±23	1.0±2.3

Transfection efficiency and cytotoxicity

**Figure 3:**
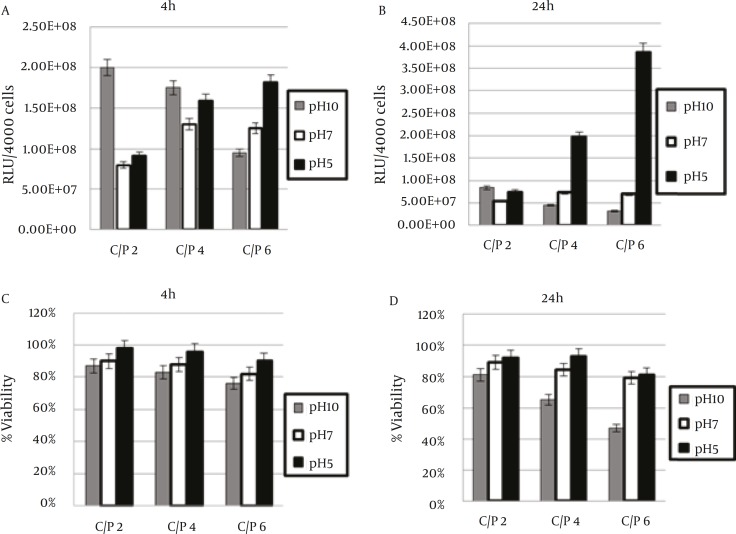
Transfection Efficiency (A and B) and Cytotoxicity (C and D) of Polyplexes in Neuro2A Cell Cultures in 96-well Trays in Different Time of Incubation (4 versus 24 hr). Luciferase Activity (A and B) and Cell Viability (C and D) Are Presented as the Mean ± SD of Triplicates

**Figure 4:**
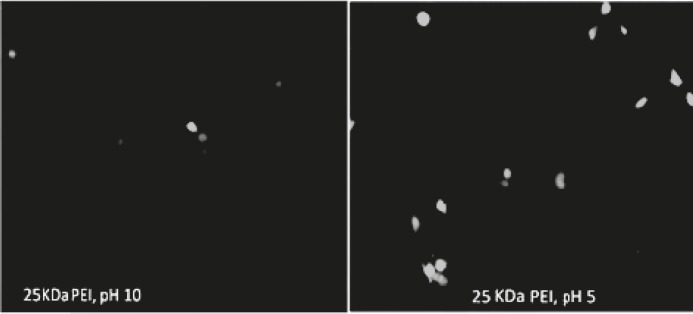
ρEGFP Expression Evaluated by Fluorescence Microscopy Following 24 hr of Incubation. Neuro2A Cells Were Transfected with PEI/ ρEGFP at (C/P) Ratio of 6.

**Figure 5:**
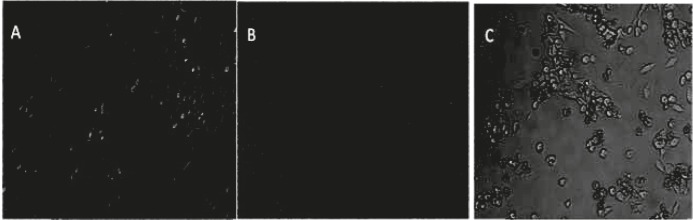
PEI Induced toxicity on Neuro2A Cells: Cells without polyplex (A), Transfected Cells with polyplexes at pH 5 (B) and transfected cells with polyplexes at pH 10 after 24 hr of incubation

The impact of the pH of PEI on the efficiency of PEI in plasmid DNA delivery was investigated on Neuro2A cell line. The cell line was transfected with 200 ng of plasmid complexed with varying amount of polymer to form different polycation/plasmid (C/P) ratios (w/w) ranging from 2/1, 4/1, and 6/1. Gene transfection efficiency was measured as luciferase enzyme activity, normalized to 4000 cells (RLU/4000 cells). The ability of PEI for pDNA delivery was analyzed at 4 and 24 hr of incubation time with cells. The efficiency of samples which were analyzed at 4 hr of incubation time have changed than those analyzed at 24 hr (Figure 3A and B). The results indicated that the transfection activities of polyplexes were higher pH 5 in 24 hr versus 4 hr incubations. The highest transfection efficiency was observed when polyplexes were prepared at pH 5 in C/P ratio of 6 and allowed to incubate with cells for 24 hr (Figure 4). The transfection of the polyplexes at pH 10 reduced after 24 hr post transfection. The cell viability of PEI against Neuro2a cells was carried out using the MTT colorimetric assay. The polyplexes were prepared at the same C/P ratio used in transfection experiments. Cytotoxicity of vectors increased with increasing in either C/P ratios or time of incubation (4 versus 24 hr). However, all vectors showed less cytotoxicity at lower pH. As revealed in Figure 3 C, no significant toxicity was observed except in the case of polyplexes prepared at pH of 10and 24 hr post incubation (Figure 5A, B, C). In this case, the PEI exhibited the highest toxicity at C/P ratios of 4 and 6, in which the viability of cells decreased to around 60% and 40%, respectively (Figure 3D). The other PEI samples showed the viability around 80-90% (Figure 3C and D). The PEI at pH 7 indicated irregular results. It was approximately higher than those observed for the transfection efficiency at pH 10 and approximately lower than those for pH 5.

## Discussion

No difference was observed among the PEI samples with different pH values at condensation ability and all of them were able to condense plasmid DNA at C/P ratio of 0.5. Additionally, higher amounts of polymer at various pHs had no impact on condensation ability. Furthermore, the condensation ability of PEI at acidic, neutral and basic environments was completely the same. 

At the pH of 5, the highest surface positive charge was achieved by the polyplexes at C/P ratio of 6 which showed the highest transfection efficiency 24 hr post transfection (Figure 3B). Although this positive charge on the polyplex surface is able to facilitate the association of these polyplexes with negatively charged cell membrane surfaces, it cannot induce significant cytotoxicity (Figure 3C and D). The prepared polyplexes at pH of 10 (C/P ratios of 4 and 6) demonstrated the highest positive charge and these polyplexes induced highest cytotoxicity (Figure 3 D). This finding is supported by the earlier investigations which show the relationship between the polyplex surface positive charge and cytotoxicity induced by polycation based gene carriers (11, 20). These results proved that the presence of positive charge is necessary for cell surface binding nevertheless it must be optimized to decrease its cytotoxic effects. Although the negative charged polyplexes are less toxic, they are not able to transfect cells effectively because they are unable bind to negatively charged cell membrane components such as proteoglycans (21, 22). As revealed in Table 1, the polyplexes at pH of 5 (C/P ratios of 4 and 6) showed the more appropriate size range for transfection experiments. Previously it has been proved that the best particle size range for gene delivery is up to 200-250 nm. Nevertheless size range is not the unique factor for effective gene delivery and several other factors such as zeta potential and DNA binding affinity may assign the carrier effectiveness. The size of the most effective carrier was around 200 nm with a net positive charge which indicates the significance of zeta potential beside the particle size range (23, 24). 

## Conclusion

In this study, the impact of pH on the PEI gene transfer ability and the charge density of PEI polyplexes were investigated. The highest transfection efficiency was achieved by the prepared polyplexes at pH of 5 at C/P ratio of 6 following 24 hr of incubation. In this condition, the net charge on the polyplexes was positive which may facilitate the electrostatic interaction with the negatively charged cell surfaces. The particle size of these polyplexes was around 200 nm and they showed the viability of 80%. The reduction of surface positive charge at pH 5 decreased cytotoxicity and caused increasing transfection time. According to the Proton Sponge hypothesis (25), buffering capacity plays a crucial role in the early escape of polyplexes from endosomes. The highest buffering capacity of PEI lies between pH 8 to 10 (26, 27). This study suggests that the proton sponge hypothesis may not be overall applicable for polymers with a low buffering capacity at pH 5 (28).
